# Three‐Year Recurrence in People With Diabetic Foot Ulcers and Chronic Limb Threatening Ischemia Is Comparable to Cancer

**DOI:** 10.1111/iwj.70724

**Published:** 2025-07-22

**Authors:** Natalie S. Armstrong, Alexandria A. Armstrong, Joseph L. Mills, Michael S. Conte, Tze‐Woei Tan, Richard S. Swanson, David G. Armstrong

**Affiliations:** ^1^ Johns Hopkins Bloomberg School of Public Health Baltimore Maryland USA; ^2^ The National Academy of Sciences Washington DC USA; ^3^ Department of Orthopaedics, Division of Podiatric Surgery University of Texas Health Science Center San Antonio USA; ^4^ The Division of Vascular Surgery and Endovascular Therapy, Michael E. DeBakey Department of Surgery Baylor College of Medicine Houston Texas USA; ^5^ Department of Surgery University of California san Francisco San Francisco California USA; ^6^ Southwestern Academic Limb Salvage Alliance (SALSA), Department of Surgery Keck School of Medicine of USC Los Angeles California USA; ^7^ Division of Surgical Oncology, Department of Surgery, Brigham and Women's Hospital Harvard Medical School Boston USA

**Keywords:** amputation, diabetes, diabetic foot, ischemia, remission, wound healing

## Abstract

This study aimed to compare the 3‐year recurrence rates of diabetic foot ulcers (DFU) and the rate of endovascular reintervention for chronic limb‐threatening ischaemia (CLTI) to recurrence rates of advanced‐stage cancers. We systematically collected original data reporting 3‐year DFU recurrence from studies published through 2024 and calculated a pooled mean. These findings were compared to recurrence rates for advanced breast, prostate, colorectal, and lung cancers using contemporary sources from the National Cancer Institute and American Cancer Society. CLTI reintervention data were drawn from the BEST‐CLI trial. The pooled 3‐year DFU recurrence rate was 58%, while the CLTI reintervention rate was 50%—comparable to cancer recurrence rates: breast (25%–40%), prostate (30%–40%), colorectal (30%–50%), and lung (60%–80%). Despite these comparable risks, DFU and CLTI remain underrecognized in terms of their recurrent burden on individuals, families, and health systems. The data presented here underscore the need to reframe healed DFU and post‐intervention CLTI not as an endpoint but as a remission—a state requiring structured surveillance and proactive management, much like in oncology. Developing interdisciplinary survivorship care plans for individuals with DFU and CLTI, modelled on those used in cancer care, may improve communication, enhance secondary prevention, and foster more ulcer‐free, hospital‐free, and activity‐rich days.

1

**Summary**

- Remission, not cure: Healing from a DFU or undergoing successful CLTI intervention should be considered a state of remission, not resolution, necessitating ongoing monitoring.
- High recurrence for DFU and CLTI: The 3‐year recurrence rate for diabetic foot ulcers (DFUs) is 58%, and for chronic limb‐threatening ischemia (CLTI) reintervention is 50%—rates that are comparable to or exceed those of advanced‐stage breast, prostate, colorectal, and lung cancers.
- Underrecognized burden: Despite these similarities, DFU and CLTI are underappreciated in both clinical practice and policy, often lacking structured surveillance and follow‐up protocols akin to those used in oncology.
- Call to action: Interdisciplinary survivorship care models—borrowed from oncology—could offer a valuable framework to reduce recurrence, improve quality of life, and increase ulcer‐free and hospital‐free days for individuals with DFU and CLTI.

## 2 Introduction

Lower extremity complications of diabetes continue to exact a significant burden on society. Approximately one third of people with diabetes will develop a diabetic foot ulcer (DFU) in their lifetime [1, 2]. Half of these DFUs will become infected and 20% of those infections will require hospitalisation [1, 2, 3, 4, 5]. Ultimately, approximately 20% of DFUs end in some form of amputation [1]. While interdisciplinary teams appear to play a strong role in reducing acute and long‐term risk for lower extremity complications, such teams remain the exception rather than the rule at centres worldwide [1, 6].

In 2020, we summarised data comparing diabetic foot complications to cancer [7]. Since diabetic foot ulcer (DFU) recurrence takes place following a period of remission, it would stand to reason that a comparison to similarly morbid cancers would be apt. Many patients with lower extremity ulcers have concomitant peripheral artery disease (PAD), which amplifies the risk for ulcer recurrence and limb loss; understanding of this critical association is thus timely and important [8]. Recent data on endovascular reintervention rates for CLTI enabled similar comparisons for lower extremity PAD [9].

## 3 Methods

The aims of this brief study were twofold: (1) to compare the 3‐year recurrence rate of diabetic foot ulcers (DFU) to that of advanced‐stage cancers; and (2) to compare the rate of endovascular reintervention for chronic limb threatening ischemia (CLTI) to these cancers. Of note, in this study, we define ‘remission’ as the period following the resolution (or healing) of a diabetic foot ulcer (DFU).

We identified original studies reporting DFU recurrence rates over 3 years post‐healing, published through 2024 [1, 2, 10, 11]. Inclusion criteria included prospective studies and randomised clinical trials. Cancer recurrence data were sourced from the American National Cancer Institute and American Cancer Society reports (through 2024), focusing on advanced stages to parallel clinical severity [12]. CLTI data were sourced explicitly from the BEST‐CLI trial [9].

## 4 Results

**FIGURE 1:**
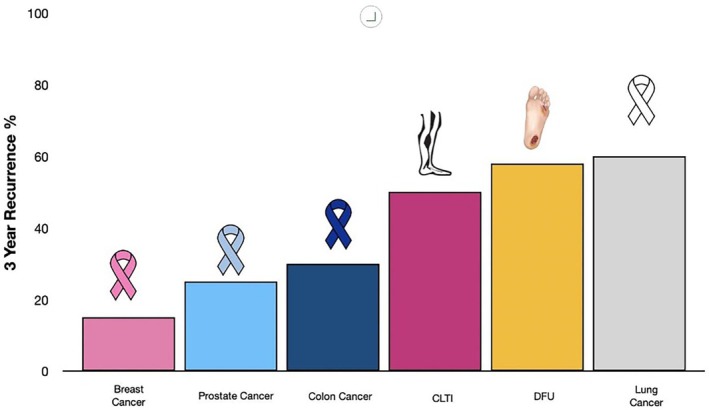
Three Year Recurrence of Diabetic Foot Ulcers, CLTI Reintervention, and Cancer in Remission. Diabetic foot ulcer recurrence and CLTI reintervention compared to cancer. DFU = diabetic foot ulcer [13, 14, 15]. CLTI—Chronic Limb‐Threatening Ischemia reintervention following endovascular intervention = 50.1 [9, 16]. Breast cancer = Triple negative breast cancer [17, 18, 19]. Prostate cancer > Stage II [17, 18, 20, 21]. Colorectal cancer ≥ Stage 2 [22]. Lung cancer (small cell lung cancer) [23]. The recurrence rates for cancers are generally based on disease‐free survival, whereas for DFUs and CLTI, they represent physical, symptomatic recurrence or need for reintervention.

Pooled data from eight prospective studies involving 1738 participants, one retrospective study with 46 participants, and nine randomised clinical trials with 636 participants in usual care groups revealed recurrence rates for diabetic foot ulcers of 42% at 1 year, 58% at 3 years, and 65% at 5 years [1, 2]. These data are illustrated in Figure 1.

The BEST‐CLI study [9], which reported outcomes for 1434 patients with CLTI randomised to either surgery or endovascular treatment (71.8% of whom had diabetes), demonstrated that outcomes after surgical bypass in patients with available ipsilateral vein conduit were superior to endovascular therapy. This conclusion was based on the risk of major adverse limb events (MALE), including major amputation, major limb reintervention (such as repeat bypass graft, graft revision, thrombectomy, or thrombolysis), or death, with rates of 42.6% for surgery versus 57.4% for endovascular therapy. In this large, prospective study, the 3‐year reintervention rate for CLTI following endovascular therapy was 50.1% [16].

The three‐year recurrence rates for certain common types of cancer, particularly for advanced stages or aggressive subtypes, while significant, are generally lower than those observed for DFU recurrence and CLTI reintervention. These 3‐year recurrence rates by cancer type are summarised as follows:

- Breast Cancer: For patients with triple‐negative breast cancer (an aggressive subtype constituting about 10%–20% of breast cancers), the three‐year recurrence rate ranges from 25% to 40%. For other advanced‐stage breast cancers (Stage III), recurrence rates at 3 years are approximately 20%–30% [17, 18, 19].
- Colorectal Cancer: Patients with Stage III colorectal cancer have three‐year recurrence rates of approximately 30%–50%. The risk increases with the number of lymph nodes involved and poor histological features [24, 25].
- Prostate Cancer: For patients with high‐risk prostate cancer (Gleason score ≥ 8, PSA > 20 ng/mL, or Stage T3‐T4), the three‐year biochemical recurrence rates range from 30%–40% [17, 18, 20, 21, 26].
- Lung Cancer: For advanced‐stage non‐small cell lung cancer (NSCLC) (Stages III and IV), the 3 year recurrence rates are as high as 60%–80%. Small cell lung cancer (SCLC), a more aggressive type, has recurrence rates exceeding 70% within 3 years [23].

These data illustrate that the three‐year recurrence rates for DFUs and reintervention rates for CLTI are comparable to, and in many cases exceed, the recurrence rates of high‐grade cancers.

For both cancer and lower extremity manifestations of diabetes and PAD, these data highlight the importance of implementing strategies to effectively monitor, detect and address disease recurrence. With respect to lower extremity complications of diabetes, however, available guidance is frequently neither available nor as well articulated to the patient or clinician.

## 5 Discussion

Despite their substantial burden on patients, families, and health systems, the high likelihood of DFU recurrence and need for CLTI reintervention remain under‐recognised in clinical practice and policy, partly due to the need for serial surveillance and the often subclinical presentation (asymptomatic) of early recurrence [27, 28, 29]. The data reported in this manuscript suggest that refocusing efforts to communicate this morbidity, just as we do with cancer, may help to reframe its importance to our patients, the rest of the health care community, and policymakers. This idea has caught the attention of leaders in the limb preservation community who have given structure to this potential dissemination [30].

Development of survivorship care plans has become a standard component of comprehensive cancer care, particularly within interdisciplinary teams managing breast, colorectal, and prostate cancers [31, 32]. Studies have shown that structured survivorship plans—emphasising coordinated follow‐up, lifestyle modification, and psychosocial support—are associated with improved patient satisfaction, better adherence to preventive care, reduced hospital readmissions, and in some cases, enhanced long‐term survival.

## 6 Conclusion

Drawing on this model, a structured remission care plan for individuals with diabetic foot ulcers (DFU) and CLTI is not only appropriate but essential [1, 2, 33, 34]. Such a plan should emphasise surveillance, risk mitigation, and patient‐centred education during periods of remission—when recurrence risk remains high yet often unrecognised. By mirroring the successful structure of oncology survivorship programmes, limb preservation teams may be empowered to help patients achieve more ulcer‐free, hospital‐free, and activity‐rich days in remission, while addressing the broader psychological and social impacts of chronic limb‐related disease.

## Conflicts of Interest

The authors declare no conflicts of interest.

## Data Availability

The data that support the findings of this study are available from the corresponding author upon reasonable request.
